# Supplementary material for: Shared and Specific miRNA-mRNA in Human and Murine Acute Kidney Injury
Source: bioRxiv. 2026 Oct 31:2026.10.30.762662. Preprint. [Version 1] doi: 10.64898/2026.10.30.762662 (PMC13734144; doi:10.64898/2026.10.30.762662)
Supplement: Supplementary file 1 [file NIHPP2026.10.30.762662v1-supplement-10.pdf]

bioRxiv preprint doi: https://doi.org/10.64898/2026.10.30.762662; this version posted October 31, 2026. The copyright holder for this preprint (which was not certified by peer review) is the author/funder, who has granted bioRxiv a license to display the preprint in perpetuity. It is made available under a CC-BY 4.0 International license.

Supplemental Figure 1. Kidney expression of canonical renal cell and acute tubular injury markers across murine AKI models. Heatmap showing expression of selected canonical renal cell markers and acute tubular injury markers across kidney samples, ordered by treatment group. Expression is shown as log2 counts per million (CPM). IRI, ischemia-reperfusion injury; CLP, cecal ligation and puncture; LPS, lipopolysaccharide; CPM, counts per million.

Supplemental Figure 2. Shared and model-specific kidney transcriptional responses across murine AKI models. (A) Volcano plot of kidney total RNA differential expression in LPS versus control. (B) GO Biological Process terms enriched among LPS-specific kidney total RNA DEGs. (C and D) UpSet plots of upregulated (C) and downregulated (D) kidney total RNA DEGs shared across AKI models. (E) Expression of representative model-specific genes (*Vim*, IRI; *Pfkfb3*, CLP; *Irf7*, LPS) across treatment groups. Expression is shown as log2 CPM; boxes indicate median and interquartile range, and points represent individual samples. DEG cutoffs as previously described in Figure 2 legend. IRI, ischemia-reperfusion injury; CLP, cecal ligation and puncture; LPS, lipopolysaccharide; GO, Gene Ontology; DEG, differentially expressed gene; CPM, counts per million.

Supplemental Figure 3. Overlap of differentially expressed kidney miRNAs across murine AKI models. (A) UpSet plot showing intersections of upregulated kidney miRNAs across IRI, CLP, and LPS models. Set sizes are indicated in parentheses. (B) Number of downregulated kidney miRNAs by AKI model; downregulated miRNAs were identified only in CLP, precluding intersection analysis. Differentially expressed miRNAs were defined as previously described in Figure 3 legend. IRI, ischemia-reperfusion injury; CLP, cecal ligation and puncture; LPS, lipopolysaccharide; FDR, false discovery rate.

Supplemental Figure 4. Biological processes enriched among predicted miRNA target modules in experimental AKI. Gene Ontology (GO) Biological Process enrichment of predicted target modules for mmu-miR-223-3p in IRI (A), mmu-miR-692 in CLP (B), and mmu-miR-155-5p in LPS (C). Up to 6 enriched terms are shown; bars indicate the number of module genes annotated to each term. No significantly enriched GO Biological Process terms were identified for the mmu-miR-223-3p target module (A). Enrichment was performed using the complete predicted target modules, with all genes detected in the integrated single-cell dataset as the background. IRI, ischemia-reperfusion injury; CLP, cecal ligation and puncture; LPS, lipopolysaccharide; GO, Gene Ontology.

Supplemental Figure 5. Shared and model-specific blood mRNA responses across murine AKI models. (A) Volcano plot of blood total RNA differential expression in IRI versus control. (B) GO Biological Process terms enriched among IRI-specific blood total RNA DEGs. (C and D) UpSet plots showing intersections of upregulated (C) and downregulated (D) blood total RNA DEGs across IRI, CLP, and LPS models. (E) Expression of representative treatment-

bioRxiv preprint doi: https://doi.org/10.64898/2026.10.30.762662; this version posted October 31, 2026. The copyright holder for this preprint (which was not certified by peer review) is the author/funder, who has granted bioRxiv a license to display the preprint in perpetuity. It is made available under a CC-BY 4.0 International license.

specific genes (*Ltc4s*, IRI; *Mpo*, CLP; *Upp1*, LPS) across treatment groups. Expression is shown as log2 CPM; boxes indicate median and interquartile range, and points represent individual samples. DEGs were defined as previously described in the Figure 2 legend. IRI, ischemia-reperfusion injury; CLP, cecal ligation and puncture; LPS, lipopolysaccharide; GO, Gene Ontology; DEG, differentially expressed gene; CPM, counts per million.

Supplemental Figure 6. Shared and model-specific blood miRNA expression across experimental AKI models. (A and B) UpSet plots showing intersections of upregulated (A) and downregulated (B) differentially expressed blood miRNA across IRI, CLP, and LPS models. Set sizes are indicated in parentheses. (C) Heatmap of treatment-specific blood miRNA, ordered by treatment group and shown as row-scaled z scores, ranked by absolute fold change; all available markers are shown when fewer than 10 were identified. DE miRNA cutoffs previously defined in Figure 3 legend. IRI, ischemia-reperfusion injury; CLP, cecal ligation and puncture; LPS, lipopolysaccharide; DE, differentially expressed; FDR, false discovery rate.

Supplemental Figure 7. Murine AKI signatures align with a shared transcriptomic axis in human AKI. (A) PCA of whole-blood total RNA from 48 KPMP participants enrolled in the AKI cohort, colored by tubular casts on biopsy (absent, n=8; present, n=26; not adjudicated, n=14). Arrows indicate the 10 genes with the largest PC1-PC2 loadings. (B-D) Samples colored by Spearman correlation with murine blood signatures for (B) CLP, (C) IRI, and (D) LPS. PCA was performed on sex-adjusted log2 CPM across 15,681 filtered genes. Top panels show centered, unscaled PCA (PC1, 20.5%; PC2, 7.4%); bottom panels show PCA after scaling genes to unit variance (PC1, 25.7%; PC2, 11.4%). Correlations with all three murine models varied predominantly along PC1 and were similar with and without scaling. Each point represents one participant.

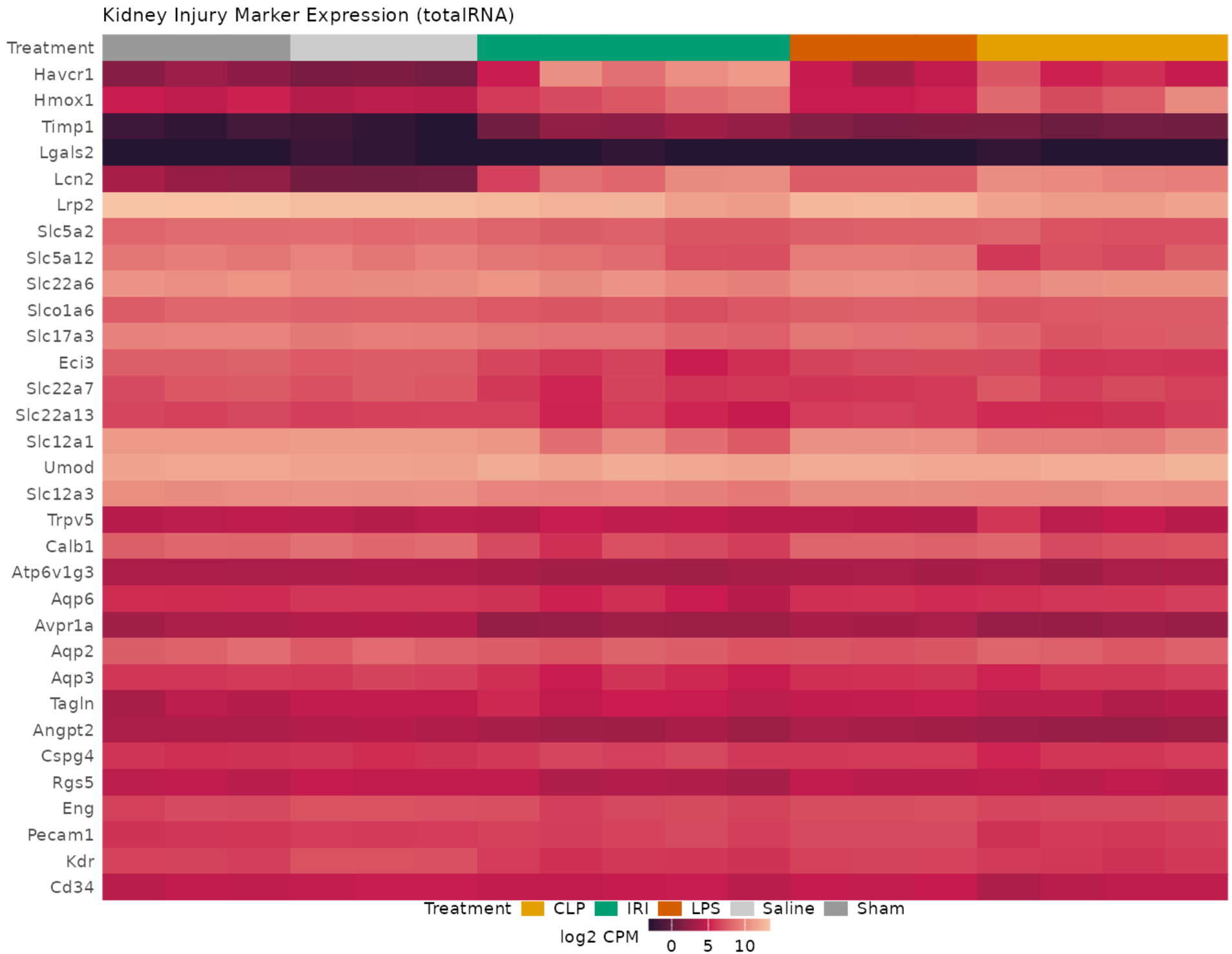

Supplemental Figure 1

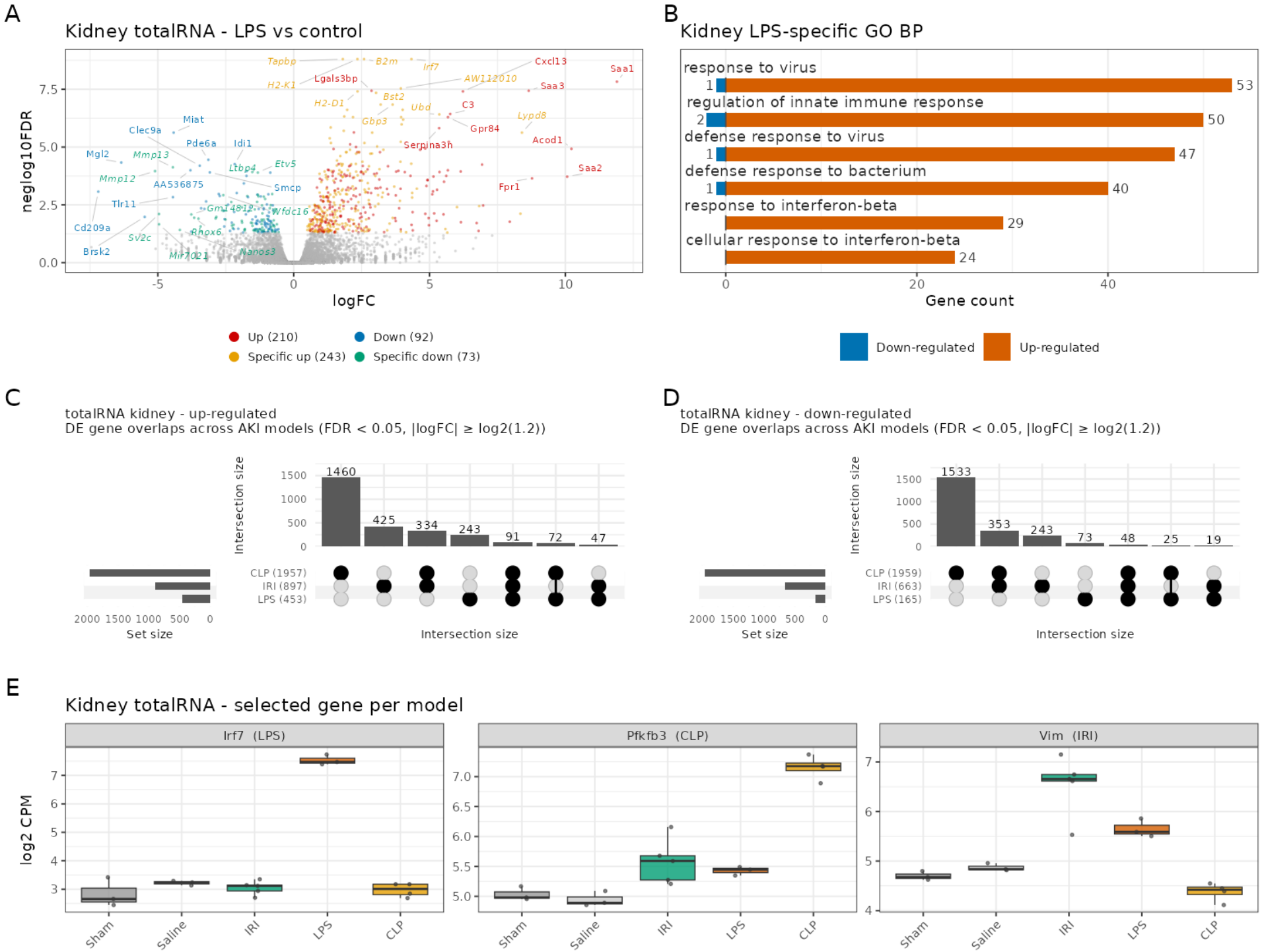

Supplemental Figure 2

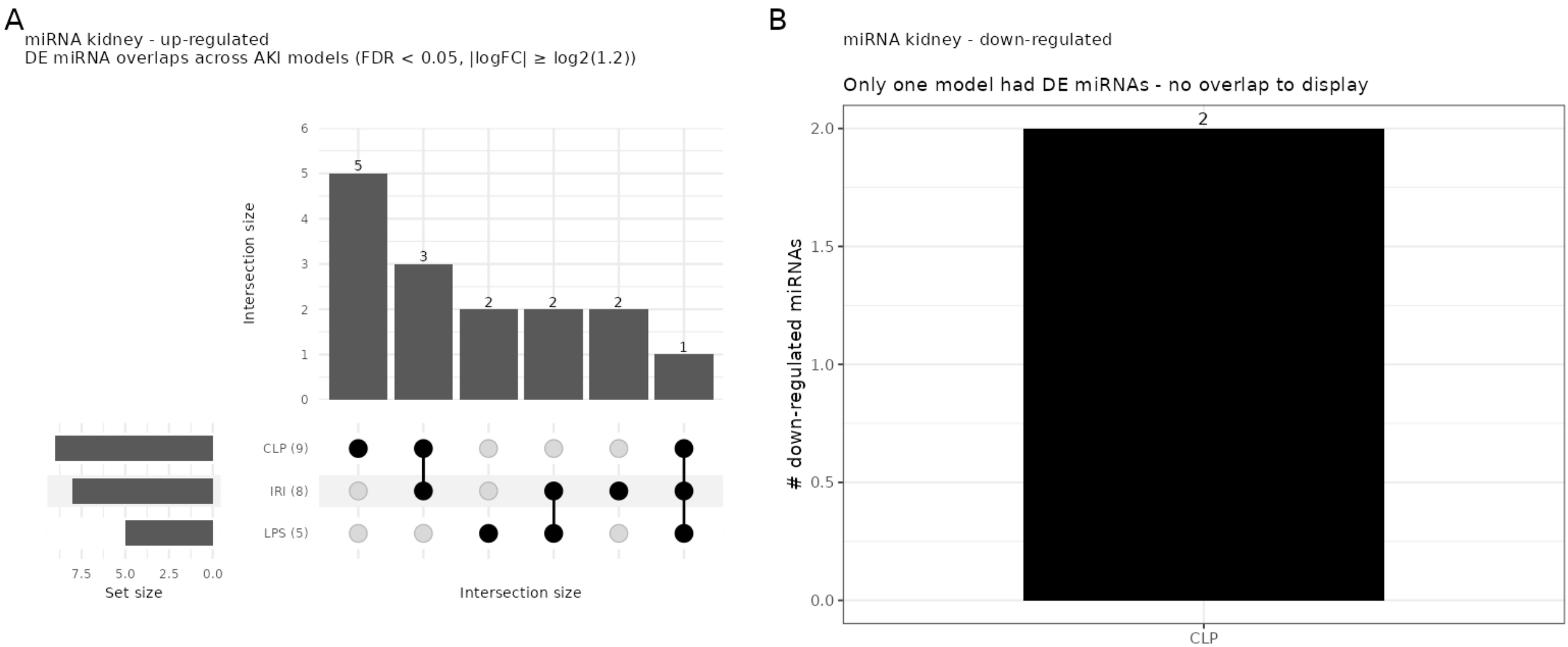

Supplemental Figure 3

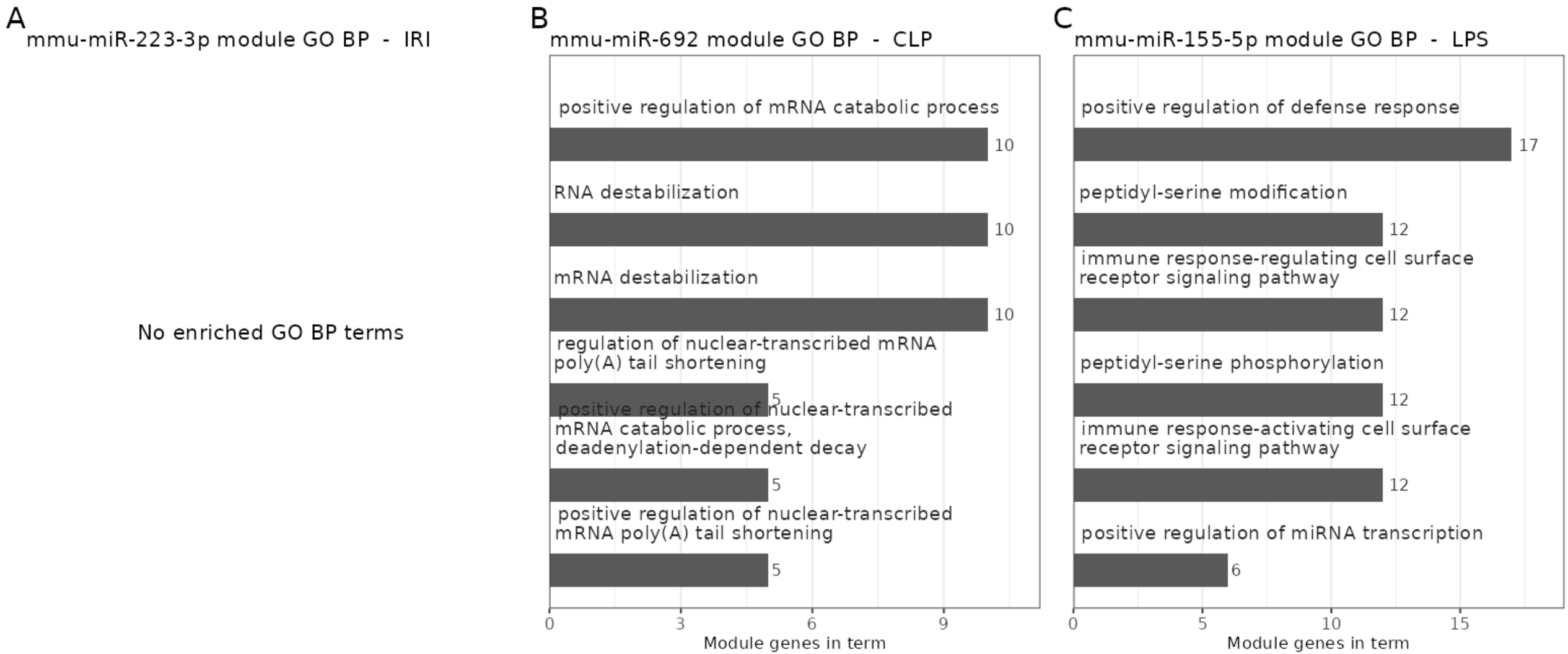

Supplemental Figure 4

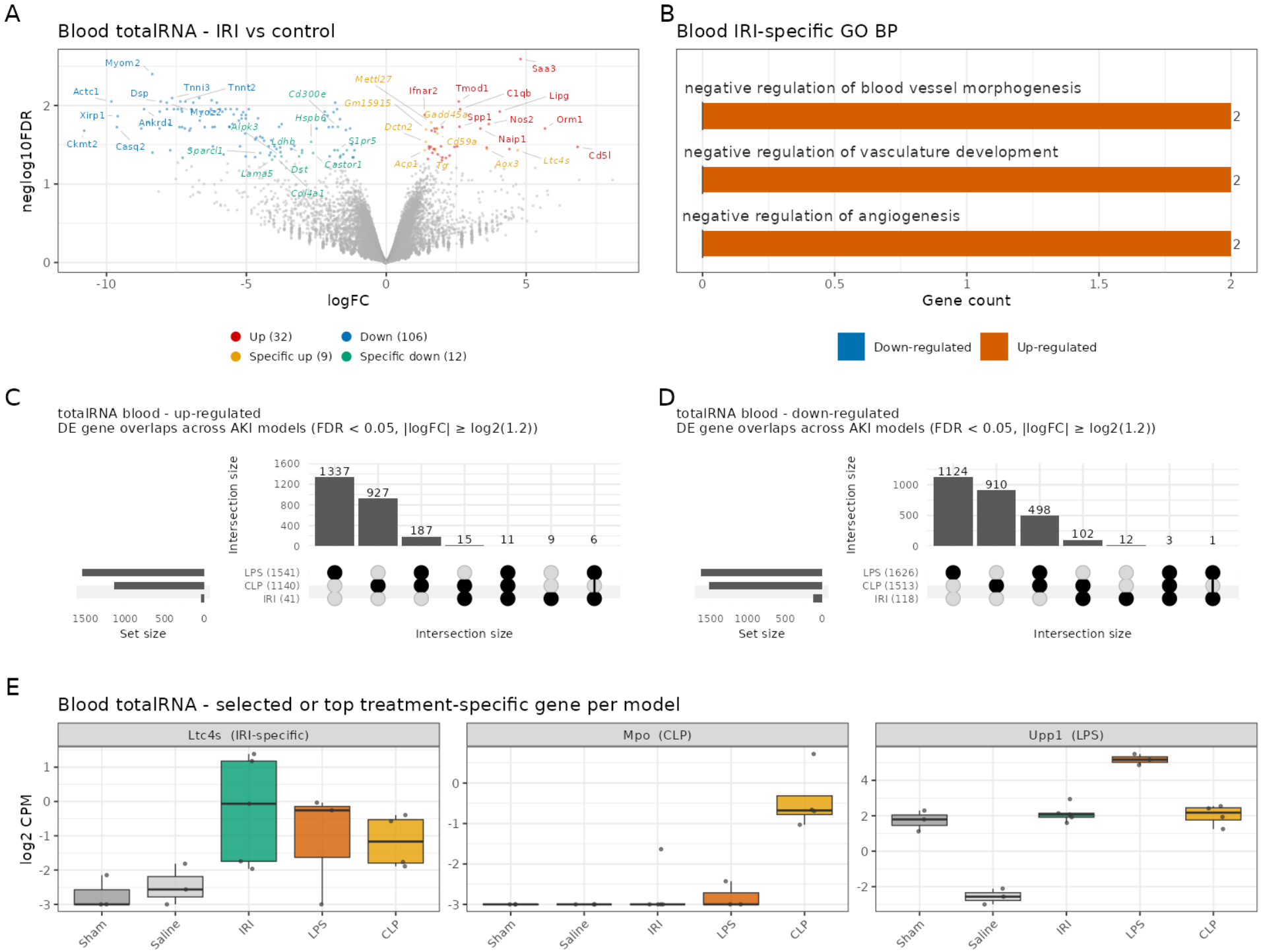

Supplemental Figure 5

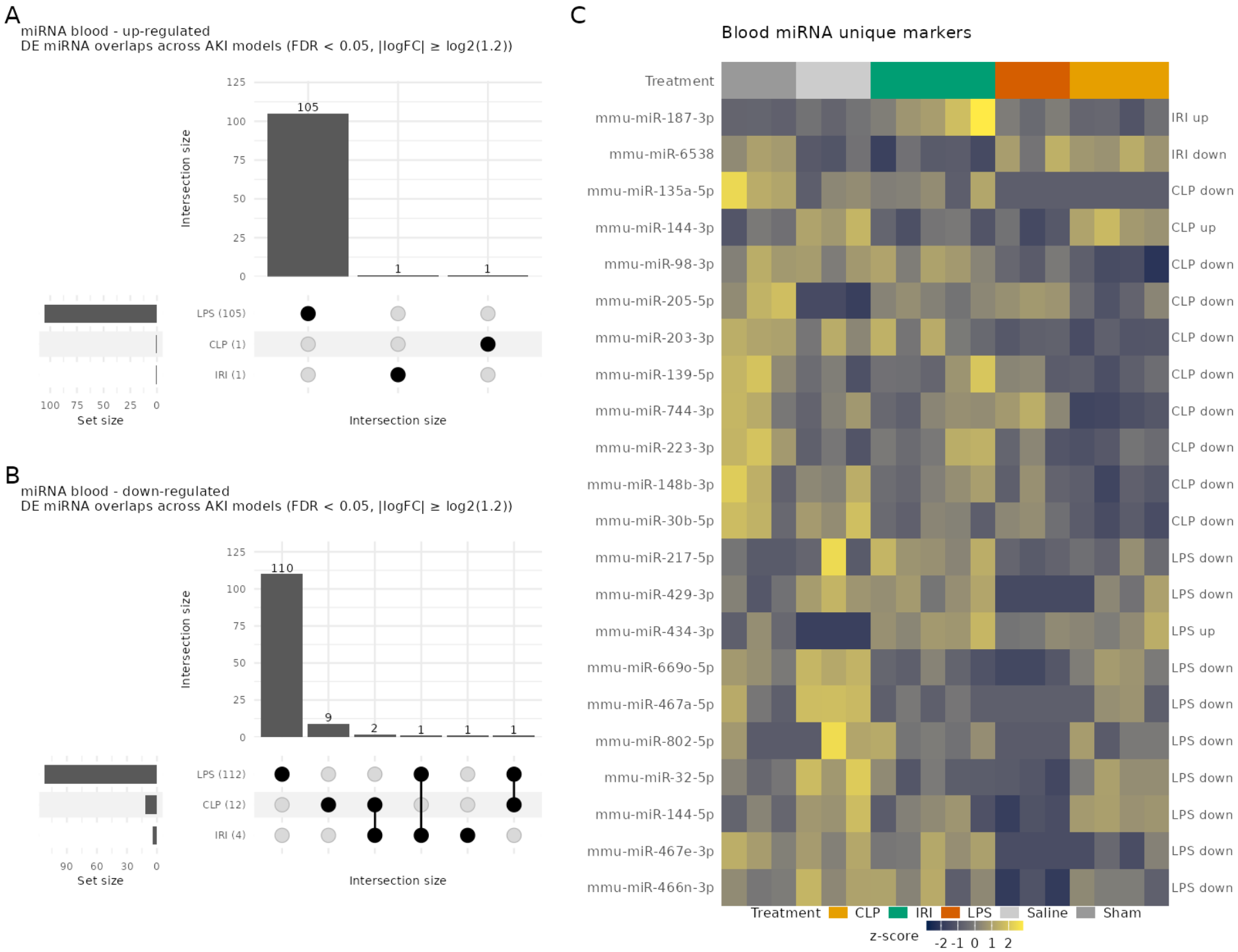

Supplemental Figure 6

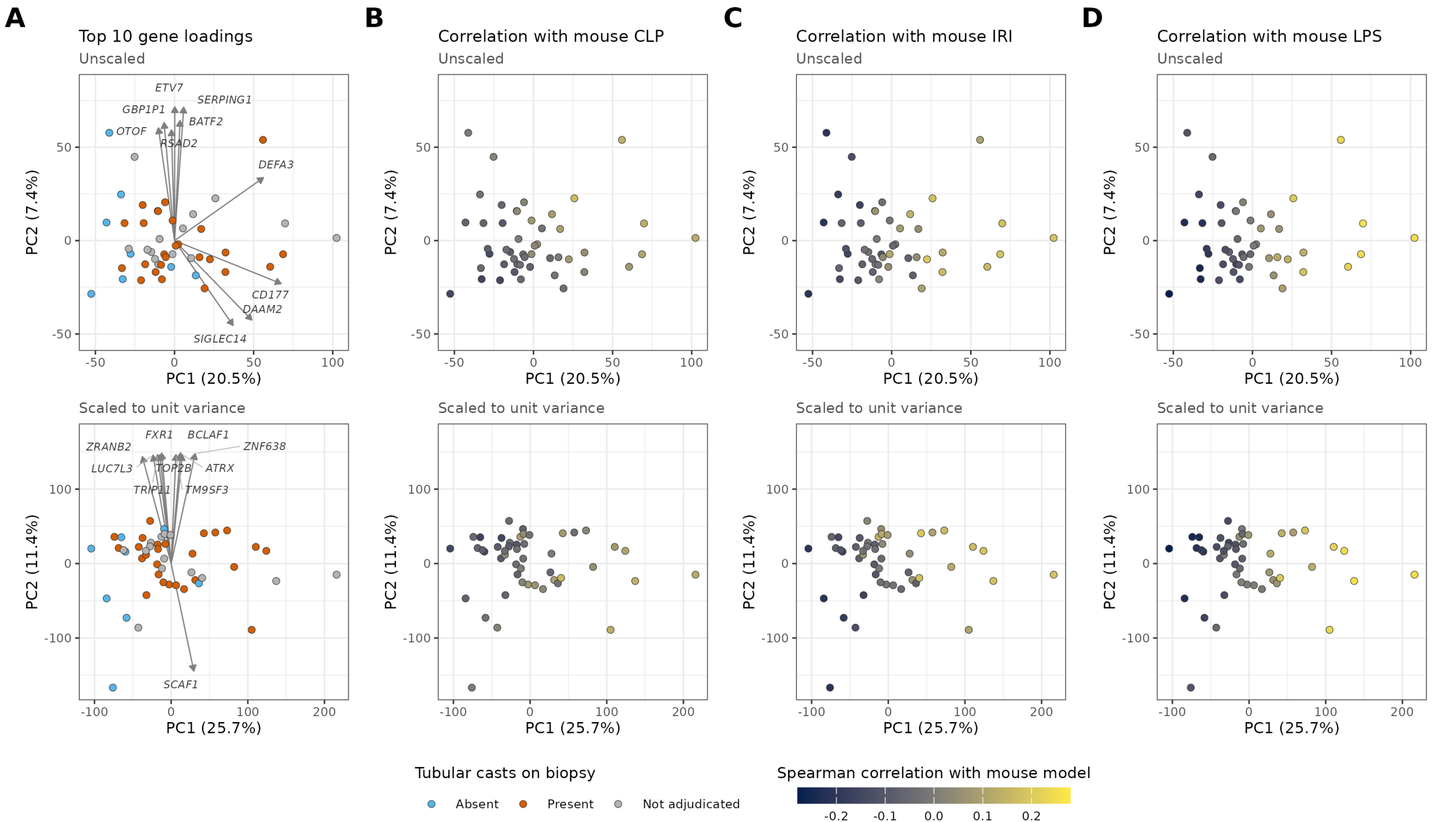

Supplemental Figure 7
